# Reconstruction with 3D-printed prostheses after type I + II + III internal hemipelvectomy: Finite element analysis and preliminary outcomes

**DOI:** 10.3389/fbioe.2022.1036882

**Published:** 2023-01-09

**Authors:** Zehao Guo, Yongjun Peng, Qiling Shen, Jian Li, Peng He, Peng Yuan, Yulei Liu, Yukang Que, Wei Guo, Yong Hu, Shenglin Xu

**Affiliations:** ^1^ Department of Orthopedics, The First Affiliated Hospital of Anhui Medical University, Hefei, Anhui, China; ^2^ Musculoskeletal Tumor Center, Peking University People’s Hospital, Beijing, China

**Keywords:** 3D-printed prosthesis, hemipelvic reconstruction, finite element analysis, sacroiliac joint, clinical outcomes

## Abstract

**Background:** Prosthetic reconstruction after type I + II+ III internal hemipelvectomy remains challenging due to the lack of osseointegration and presence of giant shear force at the sacroiliac joint. The purpose of this study was to evaluate the biomechanical properties of the novel 3D-printed, custom-made prosthesis with pedicle screw–rod system and sacral tray using finite element analysis.

**Methods:** Four models that included one intact pelvis were established for validation. Forces of 500 N and 2,000 N were applied, respectively, to simulate static bipedal standing and the most loaded condition during a gait cycle. Biomechanical analysis was performed, and the results were compared; the preliminary outcomes of four patients were recorded.

**Results:** For the reconstructed hemipelvis, stress was mainly concentrated on the sacral screws, bone–prosthesis interface, and upper endplate of the L5 vertebra. The optimization of the design with the sacral tray structure could decrease the peak stress of the sacral screws by 18.6%, while the maximal stress of the prosthesis increased by 60.7%. The addition of the lumbosacral pedicle–rod system further alleviated stress of the sacral screws and prosthesis by 30.2% and 19.4%, respectively. The site of peak stress was contemporaneously transferred to the connecting rods within an elastic range. In the retrospective clinical study, four patients who had undergone prosthetic reconstruction were included. During a follow-up of 16.6 ± 7.5 months, the walking ability was found preserved in all patients who are still alive and no prosthesis-related complications had occurred except for one hip dislocation. The Musculoskeletal Tumor Society (MSTS) score was found to be 19.5 ± 2.9.

**Conclusion:** The novel reconstructive system yielded favorable biomechanical characteristics and demonstrated promising preliminary outcomes. The method can be used as a reference for reconstruction after type I + II + III hemipelvectomy.

## 1 Introduction

The pelvic girdle is a common location for up to 15% of primary malignant bone tumors and ranks third among bone metastasis sites (Singh et al., 2016). After resection of peri-acetabular lesions, the utilization of metallic prostheses has been advantageous. Better functional recovery could be achieved through flexible adjustment of the osteotomy plane of the iliac bone during operation (Sun et al., 2011). The residual ilium can also provide a reliable bony anchorage for prostheses, which is conducive for mechanical fixation. However, when lesions invade most of region I—according to the Enneking classification (Enneking and Dunham, 1978)—and the sacroiliac joint due to the resection of the iliac wing, prostheses can only be fitted and fixed to the sacrum. The sacroiliac joint is vulnerable to shear loading on account of the predominantly flat surface. Simple screw fixation can lead to screw breakage, and the incidence of a prosthesis loosening and moving cephalad is extremely disastrous (Zhao et al., 2018). At the same time, the loss of the iliac wing makes it difficult to precisely relocate the acetabulum for the complete removal of anatomical landmarks. The excessive shift of the rotation center increases the risk of dislocation (Wang et al., 2021).

With the development of computer-aided design (CAD) and computer-aided manufacturing (CAM) techniques, prostheses can be customized with special references to individualized bone defects. 3D printing–assisted tumor resection and reconstruction have unique advantages over traditional techniques in this area with complex spatial geometry. The 3D-printed porous contact surface overcomes the shortcomings of traditional manufacturing techniques such as low porosity and uncontrollable parameters and has been proven to be beneficial to bone ingrowth and osseointegration in achieving biological fixation and long-term stability (Wang et al., 2018). Under the guidance of 3D-printed, patient-specific instrumentation, the screw trajectories can be transposed during surgery in accordance with a preoperative design, making the procedure safer.

In matching the prosthesis design to the residual pelvis, the continuity of load transfer is restored. Studies on the effectiveness of fixations and biomechanics of the pelvis are rare. It is not only difficult but sometimes also impossible to appraise stress distribution through the joint structures under different loading and boundary conditions during experimentation or through simplified mathematical models (Zanetti et al., 2012). Finite element (FE) models can be constructed for specific patients by gathering geometric and material properties from patients’ own radiographic images. The FE analysis has been widely used in the biomechanical study of implants and provides an effective *in silico* approach to gather information on static and dynamic responses (Liu et al., 2016).

In the literature, we however found a wide spectrum of implant designs across patients who had received pelvic reconstructions (Wang et al., 2019b; Peng et al., 2020; Wang et al., 2020; Wu et al., 2021; Zhu et al., 2021). An optimal design for a prosthesis with good biomechanical properties is still unclear. This study aims to 1) construct FE models before and after prosthetic reconstruction that simulate human reality; 2) analyze the biomechanical properties of prostheses and the effects of the pedicle screw–rod system and sacral tray on load transmission; and 3) observe the efficiency and stabilization of prostheses for tumor-induced hemipelvic defects in four patients and provide references for routine clinical use.

## 2 Materials and methods

### 2.1 Prosthesis design and finite element model construction

**FIGURE 1:**
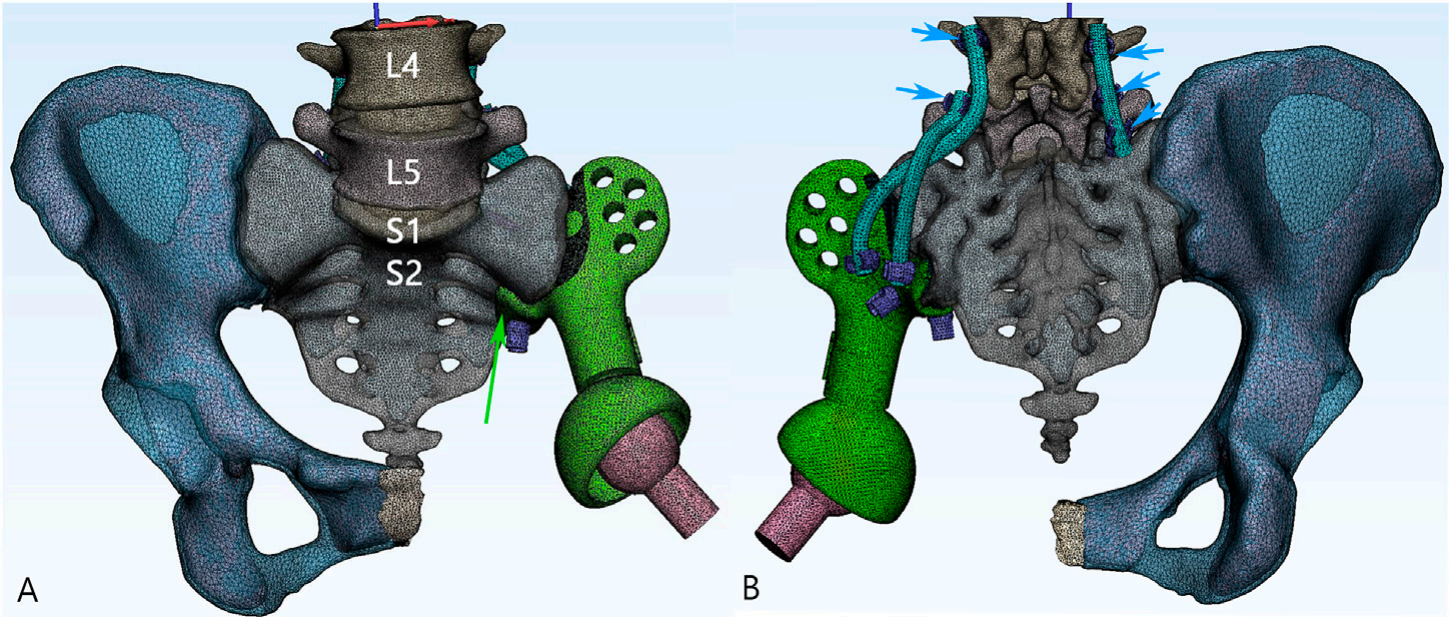
3D model of the reconstructive system. **(A)** The anteroposterior view of the model. Green: the main body of the prosthesis. Pink: the artificial femoral head. Green arrow: the sacral tray structure. **(B)** The posterior aspect of the model. The screws being fixed on the prosthesis are defined as sacral screws (purple). Lumbosacral rods (blue) are fixed with five pedicle screws (short blue arrow).

We chose one patient with the highest weight and body mass index (female, 54 years old, 158 cm in height, 74 kg in weight) diagnosed with chondrosarcoma to be the subject of the FE analysis. Digital Imaging and Communications in Medicine (DICOM)–based CT scans for the pelvic region at .625 mm slice thickness were imported into Mimics 19.0 software (Materialise Inc., Leuven, Belgium) to reversely construct a 3D model. The processed images of contralateral hemipelvis were used to create a mirror model. Only a part of the proximal femur was preserved. The thickness of the cortical bone was assumed to be around 2 mm throughout (Anderson et al., 2005). As a comparative study, the cortical thickness of all the models was considered to be the same. The intervertebral and interpubic discs were constructed by filling up the spaces between the related bony surfaces, referring to the gray scales and anatomic position. Next, the model was processed for surfacing smoothing. The model was then imported into the Magics software (Materialise Inc., Leuven, Belgium) to reconstruct the hemipelvic prosthesis, screws, and rods. The artificial femoral prosthesis was simplified as a sphere head and cylinder neck for the biomechanical analysis of the lower extremity being omitted in this study. The design stemmed from the concept of a metallic metal prosthesis that could connect the spine and ipsilateral lower extremity to rebuild load transfer through a bilateral rod–screw system. The sacral tray was incorporated to be fully fitting with the ventral sacrum surface to mitigate the shear force imposed on the screws. The length and trajectory of the polyaxial screws were designed in advance to avoid penetration into the sacral canal or anterior neurovascular plexus. The screws passed through the L5, S1, and S2 vertebrae to bear the vertical stress from different directions and exert compressive force to facilitate osteogenesis. One screw was designed to be inserted through the sacral tray up to the sacral wing to provide angular stability (Figure 1). Four groups of finite element models were built: the intact pelvis (Control), the initially designed prosthesis with only three sacral screws for fixation (Model 1), modified prosthesis with the addition of the sacral tray (Model 2), and reconstructed hemipelvis with the pedicle screw–rod system and sacral tray (Model 3), i.e., the definitive design adopted in patients.

### 2.2 Material parameter settings and boundary loading force conditions

**FIGURE 2:**
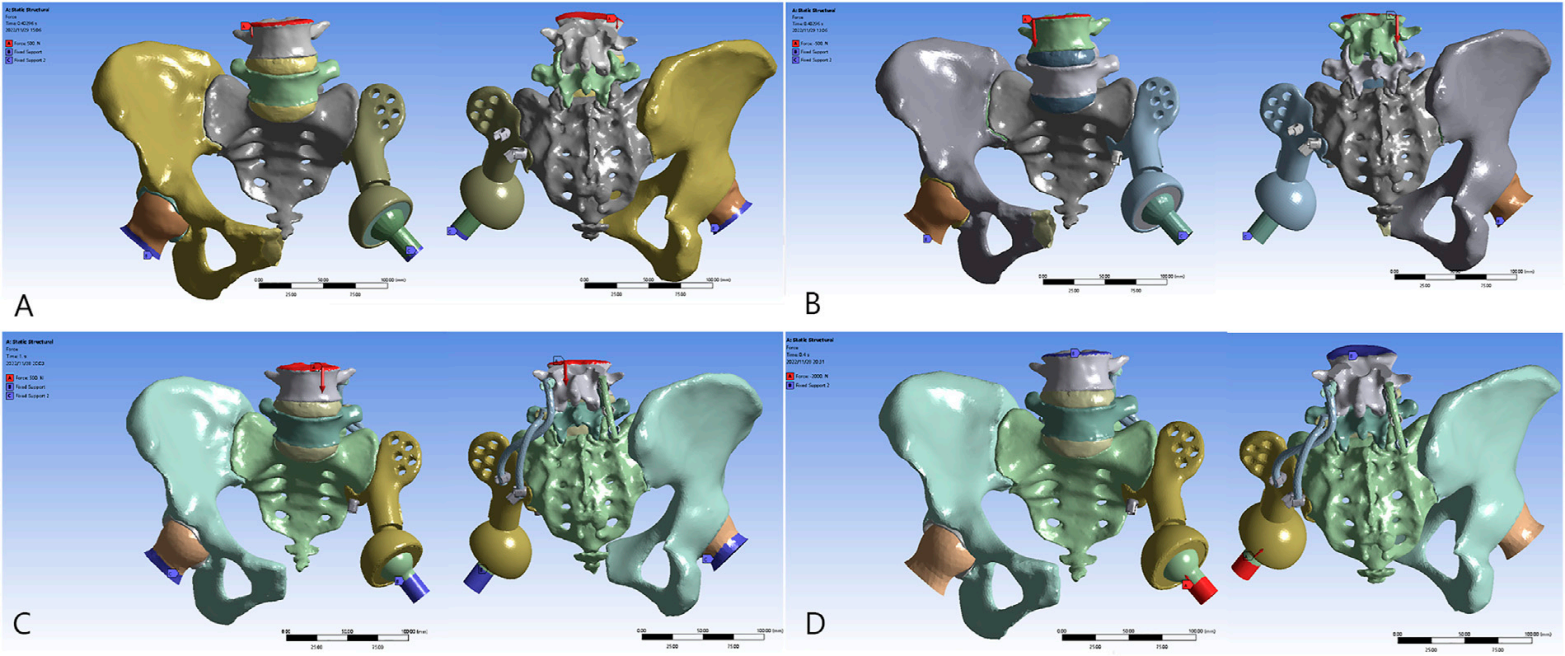
Reconstructive models for FE analysis: **(A)** Model 1_B, **(B)** Model 2_B, **(C)** Model 3_B, and **(D)** Model 3_E.

3D models in stereolithography (STL) format were imported into Ansys Mechanical 2022 R1 software (Ansys, Canonsburg, PA, United States) for further analysis. The mesh quality was checked and optimized, and the linear 8-node hexahedral mesh (SOLID185) was generated according to the surface mesh (Table 1). It was assumed that the bone, annulus fibers, nucleus pulposus, and metal were all continuous, isotropic, and uniform linear elastic materials. The material characteristics of various structural materials are shown in Table 2. A vertical downward load of 500 N was imposed on the surface of the L4 vertebra to simulate the gravity of the body in static poses. The four models were subjected to bipedal standing (Control, Model 1–3_B), and the femoral neck of the healthy and reconstructed sides was constrained symmetrically. An axial load of 2,000 N was imposed on the center of the artificial acetabulum in Model 3 (Model 3_E) to mimic the most loaded circumstance in one gait cycle (Bergmann et al., 2001; Wang et al., 2022; Zhou et al., 2022). In terms of boundary conditions, the area of the upper surface of the L4 vertebral body was considered to be fully bonded (Figure 2). The contact was set as bonded. The von Mises stress was selected as the main parameter for biomechanical evaluation.

**TABLE 1:** The number of elements and nodes for the models.

Model	Node	Element
Control	2392221	1320856
1	2517862	1396130
2	2436351	1348870
3	1867529	1024768

**TABLE 2:** The material characteristics of various components.

Component	Young’s modulus (MPa)	Poisson’s ratio
Cortical bone (Liu et al., 2019)	17,000	.3
Cancellous bone (Liu et al., 2019)	150	.2
Annulus fibrosus (Shen et al., 2022)	4.2	.49
Nucleus pulposus (Shen et al., 2022)	1	.49
Ti6Al4V (Liu et al., 2019)	110,000	.3

### 2.3 Clinical study

The retrospective study identified patients subjected to type I + IIⅡ + III hemipelvic resection and 3D-printed, custom-made prosthetic implantation from January 2019 to May 2022, and four patients with modified prosthesis design (Model 3) were enrolled. Detailed prosthesis fabrication, surgical procedure, and postoperative management were the same to those described in previously published studies (Liang et al., 2017; Xu et al., 2022). Patient demographics, complications, and outcomes were collected (Table 3). Functional evaluation of the affected limb was determined by the Musculoskeletal Tumor Society (MSTS) scores at the latest follow-up (Enneking et al., 1993).

**TABLE 3:** The demographics of the four patients treated with optimized prosthesis.

Patient number	Age (years)/gender	Pathological diagnosis	Complications	Follow-up (months)	Status	MSTS-93
1	55/F	Chondrosarcoma	DWH	18	NED	16
2	53/M	Chondrosarcoma		18	AWD	23
3	53/F	Chondrosarcoma		24	NED	19
4	51/F	Chondrosarcoma	DWH	6	NED	20

DWH, delayed wound healing; NED, no evidence of disease; AWD, alive with disease.

## 3 Results

### 3.1 Validation of the developed finite element model

**FIGURE 3:**
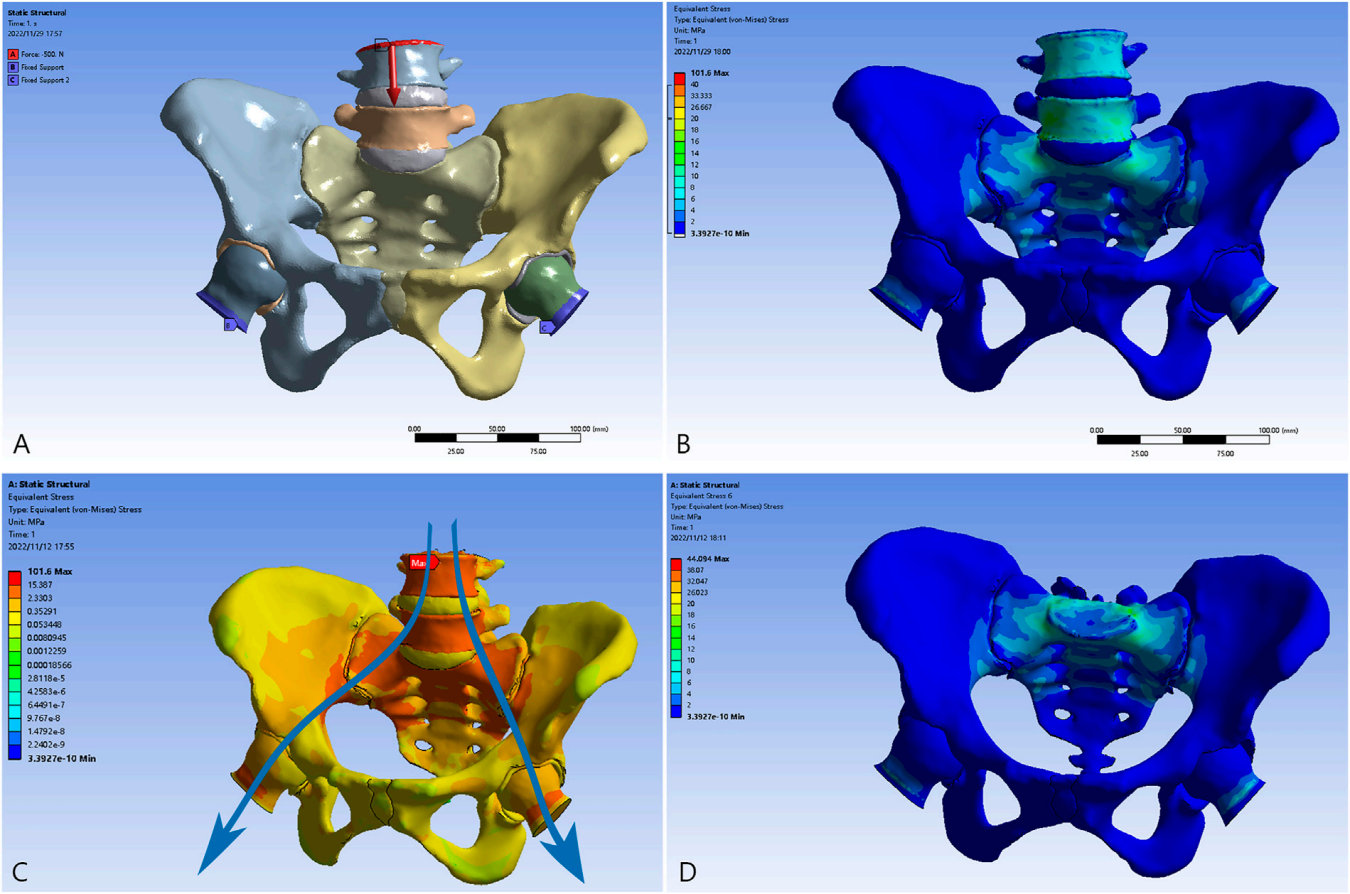
The validation model of the normal pelvis. **(A)** The bilateral proximal femurs were restrained, and the load was applied to the L4 vertebra. **(B)** The distribution of the von Mises force. **(C)** The transmission of loading stress from the trunk (blue arrows). **(D)** The distribution of the force in the pelvic ring.

Stress concentrated at the L4, L5, and S1 vertebrae and in the region around the sacroiliac joint is transferred through the arcuate line and the superior area of the greater sciatic notch to the hip joint (Figure 3), exhibiting a satisfactory bionic degree; the magnitude of stress tapers off downside the sacral midline. The ilium and ischiopubic region are in a state of relatively low stress. A maximal stress of 101.6 MPa was observed when standing in the bipedal posture. With regard to stress of the pelvic ring, a peak stress of 44.1 MPa was observed on the upper endplate of the S1 vertebra, which is consistent with intuition and previous research in distribution and scales (Iqbal et al., 2017; Liu et al., 2019; Zhou et al., 2022).

### 3.2 Stress and displacement distribution

**FIGURE 4:**
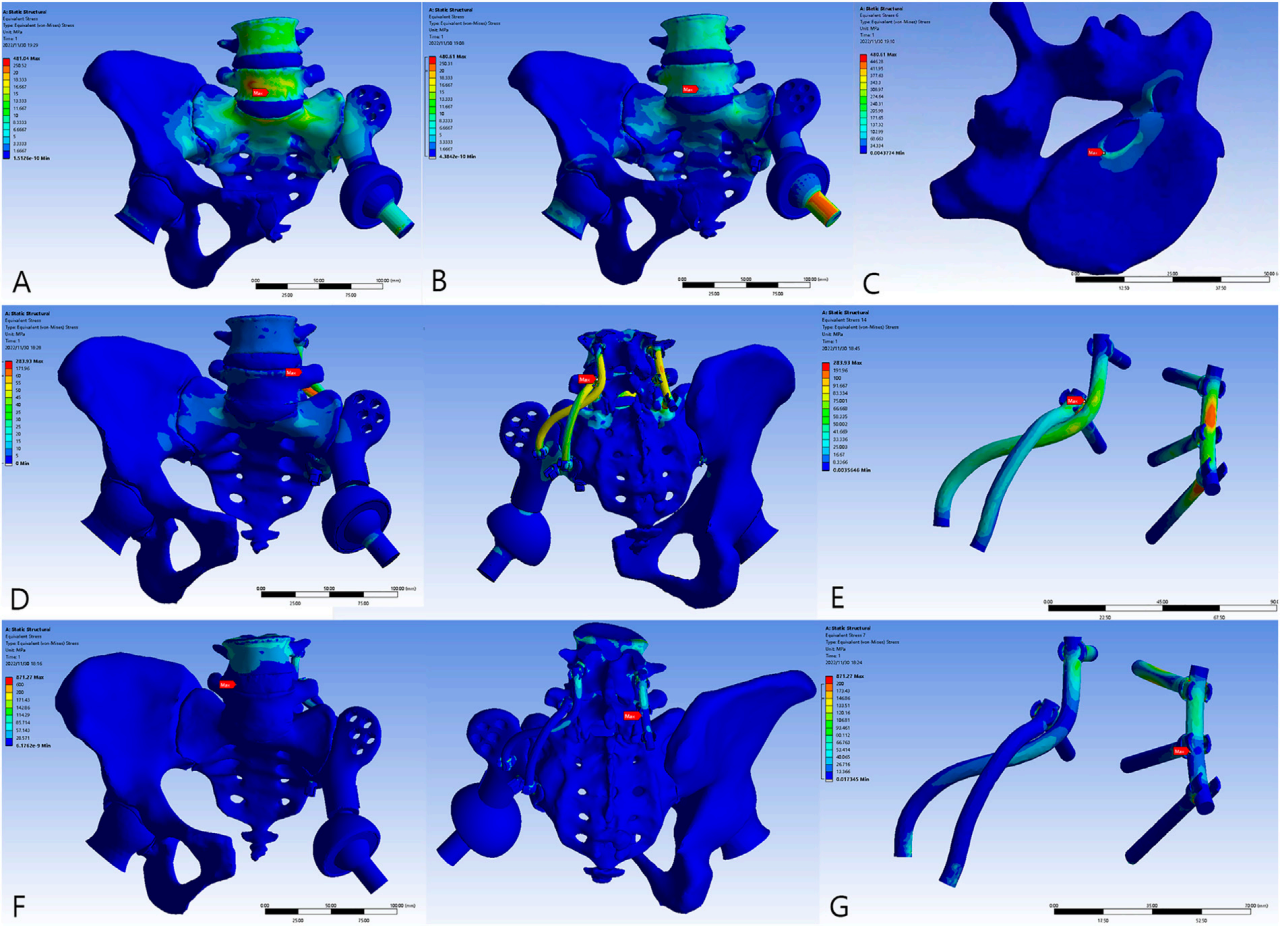
The von Mises force of the models. **(A)** Model 1_B. **(B)** Model 2_B. **(C)** The peak force of Model 2_B. **(D)** Model 3_B. **(E)** The peak force of Model 3_B at the locking site of the pedicle screw and the rod. **(F)** Model 3_E. **(G)** The peak force of Model 3_E at the pedicle screw–rod system of the healthy side.

**FIGURE 5:**
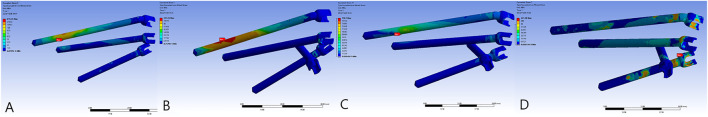
The von Mises force of the sacral screws. The three horizontal screws were L5, S1, and S2 screws from top to bottom. **(A)** Model 1_B. **(B)** Model 2_B. **(C)** Model 3_B. **(D)** Model 3_E.

**FIGURE 6:**
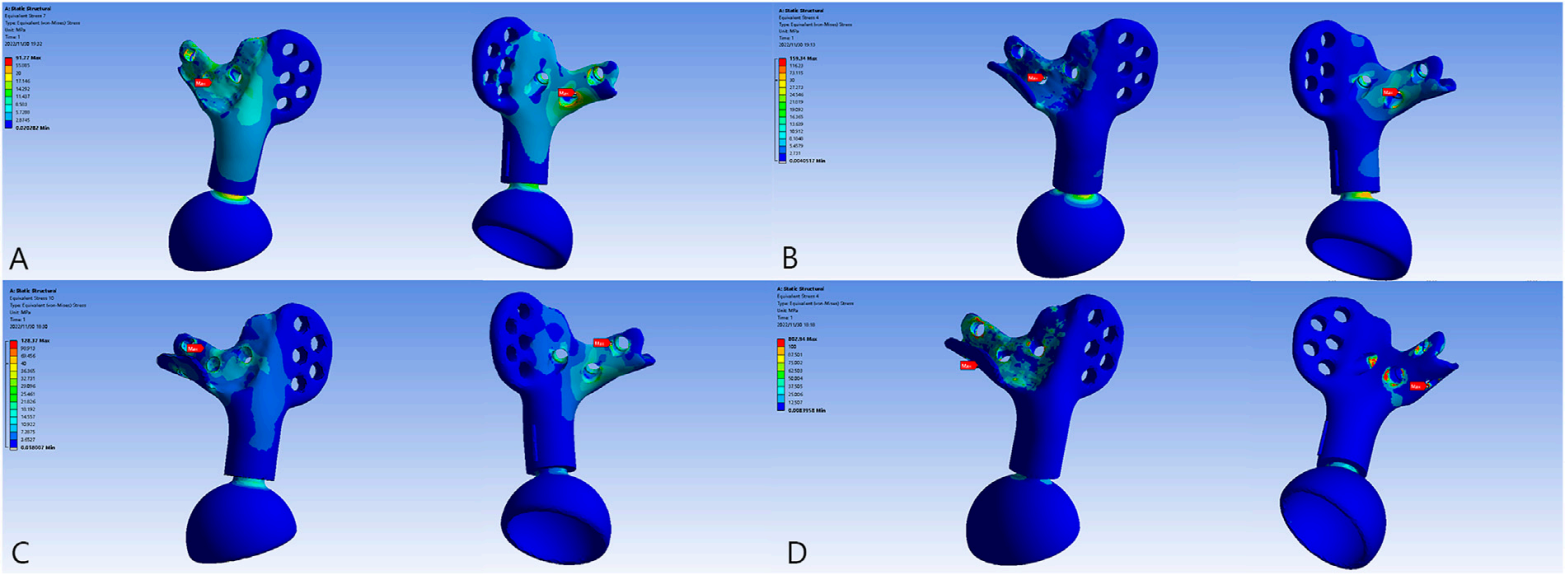
The von Mises force of the prosthesis and the force concentrated near the screw holes. **(A)** Model 1_B. **(B)** Model 2_B. **(C)** Model 3_B. **(D)** Model 3_E.

The overall distribution of the von Mises stress and deformation under vertical loads is illustrated in Figures 4–6. When compared to the native pelvis, force conduction shows a similar but less smooth tendency. Stress was concentrated at the bone–metal interface and diminished along the body of the prosthesis. In upright weight-bearing stances, there was an obvious stress concentration on the main body of the L5 screw. The addition of the sacral tray reduced maximum stress by 18.6% (from 279.45 to 227.46 MPa), while maximal deformation of the prosthesis increased from .05 to .15 mm. Peak stress of the whole model arose at the upper endplate of the L5 vertebra where the L5 screw had penetrated (481.04 MPa for Model 1_B and 480.61 MPa for Model 2_B) and maximal stress of the prosthesis was increased by 60.7% (from 99.17 to 159.34 MPa).

The introduction of the screw–rod system could further reduce stress on the sacral screws and prosthesis by 30.2% (from 227.46 to 158.70 MPa) and 19.4% (from 159.34 to 128.37 MPa), respectively. Concurrently, the aberrant stress concentration was alleviated (from 480.61 to 283.93 MPa) and occurred at the contacting site of the pedicle screw and rod. When the posture was switched to the most loaded condition during a gait cycle with one foot standing, there were extensive regions of high stress. Peak stress of the sacral screws appeared at the tail of the screws and increased to 641.28 MPa. The pedicle screw–rod system of the healthy side became the site of stress concentration (871.21 MPa).

### 3.3 Clinical results

**FIGURE 7:**
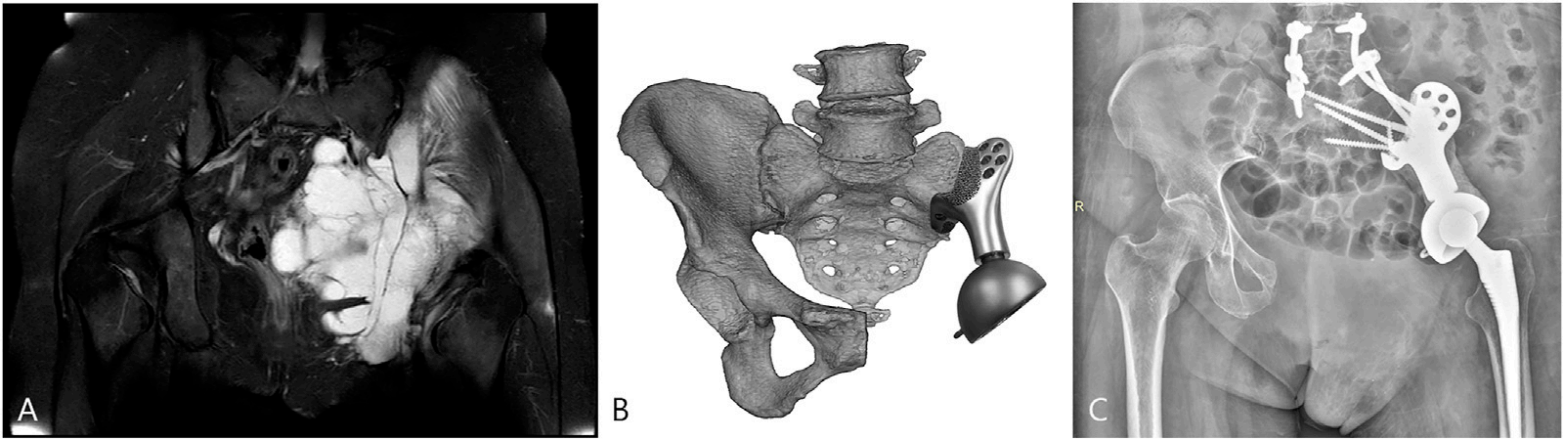
The patient (case 3) with the highest BMI was selected for FE analysis. **(A)** Preoperative magnetic resonance imaging (MRI) showed the involvement of the sacroiliac joint. **(B)** The surgery simulation that fitting the sacral tray onto the ventral sacrum. **(C)** The postoperative X-ray at the latest follow-up.

All patients were alive at the latest follow-up of 16.6 ± 7.5 months. Two patients had wound healing disturbance at the crossing site of the T-shaped incision attributable to excessive ligation of the terminal branch of the superior gluteal artery encompassing the tumor. One of the patients (case 1) received three cycles of irrigation and debridement, followed by a second-stage flap transfer. The patient experienced a recurrence at 16 months after surgery. This was followed by palliative tumor resection and implantation of radioactive ^125^I seeds. Multiple surgeries and prolonged bed rest impeded rehabilitation and resulted in inferior limb function. At 8 months after surgery, one patient (case 2) had a local recurrence which was treated with expanded resection and was diagnosed with pulmonary metastasis at 3 months before the latest follow-up. No signs of component breakage or aseptic loosening was observed (Figure 7), and the ability to ambulate was restored with a MSTS-93 score of 19.5 ± 2.9.

## 4 Discussion

Static load analysis allows the localization of stress concentration points on artificial implants under load conditions, which can be useful in evaluating implant safety and improving design, since fatigue fracture tends to occur here. In cases of prostheses of the lower extremity, the higher peak loads reached in cyclic dynamic conditions can better reflect the real situation. However, static load analysis is alone acceptable for patients who have undergone type I + II + III resection as they can only obtain an altered gait resembling claudication and may require braces or crutches to assist in long distance walking. This comparative study focused on the overall stress distribution of the reconstructive system, and screw stress was compared horizontally. Therefore, pre-strain of the screws was not considered and surface contact was defined directly in order to increase efficiency. This will be incorporated into future studies regarding the optimization of screw structures.

During hemipelvic reconstruction surgery, pure sacral screws and the utilization of the lower lumbar spine as a strut are two methods that have been widely used to prevent proximal displacement of prostheses (Wang et al., 2015; Wang et al., 2020). The nearly vertical auricular surface brings high shear force and breakage of screws is disastrous. However, it is also worth noting that the introduction of the posterior lumbosacral procedure in select patients increases operation time, surgical trauma, and blood loss. Theoretically, the firm internal fixation weakens stress stimulation at the bone contact surface, which is not conducive to osteogenesis (Liu et al., 2019).

In the absence of the lumbosacral pedicle screw system, apparent stress of 480.61 and 481.04 MPa was observed at the cortical bone of the L5 vertebra where the L5 sacral screw had penetrated (Figure 4C) as a result of the differences in texture of the bone and of titanium. Stress had been generated near the interface of the titanium alloy during transmission of force and deformation downward the spine, since the titanium alloy has a greater elastic modulus and little capacity for concession. However, in Model 3_B and Model 3_E, stress concentration could be improved dramatically (Figures 4D–G). Accordingly, after constructing the pedicle–rod system, force could be conducted through the bilateral rods. The stress distribution was therefore more even and reasonable than it would have been without spinal implants.

Unilateral solid spinal fixation increases displacement of the unaffected hemipelvis (Liu et al., 2019). Therefore, immobilizing the lumbosacral region of the healthy side could contribute to a more even displacement level, avoiding increased compensatory range of motion and secondary joint degeneration to a certain extent. In addition, bilateral fixation acts against torsional stress and provides reliable stability (Otsuki et al., 2021). Consequently, under vertical load, the pedicle screws and rods restrict the lower lumbar spine from physiological movement, causing it to endure greater stress, thereby becoming sites with peak stress in the entire reconstruction system (Figures 4D–G). As a result of the elastic modulus gap between the vertebrae and internal fixation device, stress tends to be concentrated on the cranial part of the screws and rods, thereby heralding a high risk of failure. Moreover, patients with spinopelvic fusion have a constrained posterior pelvic tilt and countervailing hip flexion from standing to sitting positions, increasing the risk of dislocation, especially in those who have undergone extensive soft tissue resection (Esposito et al., 2018). However, it should be noted that by the addition of spinal instruments, sacral screws, and lumbar screw–rods, a complementary dual fixation system is formed that allows sufficient breakage–reoperation interval for revision surgery and simplifies the procedure with a simple posterior approach. It is thought that lumbar integration increases spinal column force conduction. Although intervertebral fusion could decrease the maximum von Mises force by up to 33% (Wang et al., 2015), since extra procedures might increase blood loss and operation time significantly, only screw–rod internal fixation could provide sufficient stability both in static and dynamic conditions.

In addition to using the lumbar vertebrae as the anchor point for mechanical fixation and modifying the structural design of the prosthesis, the trajectory of the screws is also critical for maintaining stability. The articular surface area limits the sacroiliac joint being inserted with up to three or four screws. Novel bone mineral density channels have been identified for screw insertion to improve the distribution of stress, promote bone growth, and enhance the biomechanical properties of the prosthesis (Zhou et al., 2022). In our study, the orientation of the screws was more empirical. Three sacral screws were designed to bear partial longitudinal loading and impose axial compression to promote osteogenesis. The fourth screw inserted from the sacral tray was expected to maintain the stability of the prosthesis in the sagittal plane and thus be minimally stressed in the upright position. The screw–rod system reduced the average and maximal stress of the screws. For type I + II + III reconstruction, mechanical fixation failure was mostly attributed to stress concentration at the sacroiliac screws and subsequent breakage (Zhang et al., 2018). Spinal fixation was corroborated in this study to exert a protective effect on the sacral screws (from 227.46 to 158.70 MPa, Figures 5B, C) and the prosthesis (from 159.34 to 128.37 MPa, Figures 6B, C). Similarly, the novel design of the sacral tray could reduce stress of the sacral screws effectively (from 279.45 to 227.46 MPa, Figures 5A, B) which exerted a superior degree of protection when compared to the sacral hook reported previously (Wang et al., 2019a). Due to the 3D-printed, custom-made nature, the tray perfectly fitted the ventral sacrum and offered larger contact areas, which prevented concentration of stress and allowed for an additional screw to be inserted. The sacral tray structure stabilizes sacrum deformity and reduces stress on the screws, but maximum stress of the prosthesis increases (Figures 6A, B). However, this increase is acceptable, so it is a reasonable structural improvement that can bring benefits.

The reconstruction of region III was treated with benign neglect given the reconstructed weight-bearing axis. As the pelvis is a circular structure closed by amphiarthroses, there is a biomechanical interdependence between the sacroiliac joints and pubic symphysis (Ricci et al., 2020). The increased laxity of the anterior ring causes the posterior ring to experience greater stress. In extreme cases, the total resection of the pubic symphysis and strong arcuate ligament resulted in opposing horizontal displacement of the bilateral hemipelvis during the gait cycle, even under static vertical loads, such that the unaffected hemipelvis might develop instability and secondary osteoarthritis (Li et al., 2020), while the bone–prosthesis interface on the reconstructed side also suffered from additional pull-out force. Thus, a higher stability of prosthesis strengthening by spinal instruments in the posterior ring is required.

Currently, the origin of the adductor muscle is sutured to the abdominal wall muscle. The transposition leads to weak adductor strength and the limb tends to rotate externally, making it prone to anterior dislocation. The advantage of region III reconstruction is that it provides a rigid attachment for soft tissue reconstruction, which not only provides a direct barrier against pelvic viscera but also facilitates the attachment of the mesh for abdominal wall reconstruction to prevent the formation of hernias (Zhang et al., 2021). At the same time, the adductor muscles and hamstring muscles could be reattached anatomically, ensuring better functional recovery and pain improvement. However, the natural pubic symphysis permits a minute shift of 2 mm and 1° of rotation, which prevents stress fracture under peak loading (Hammer et al., 2019; Tseng et al., 2022). After rigid prosthetic reconstruction of region III, the pubic symphysis bore greater vertical deformation than the other parts (Dong et al., 2014). Sealing the pelvic ring with metallic connections such as pubic rami plates or rods restricted its physiological mobility, bringing extra force concentration and subsequent implant failure. For patients with traumatic pubic symphysis diastasis, the high rate of mechanical failure might represent a benign condition that normal motion is restored to the articulations of the pelvis (Eastman et al., 2016). Whereas the breakage of implants after type III hemipelvectomy is an allusion to the failure of anterior ring reconstruction and possibility of major revision surgery. In a retrospective, small cohort study, reconstruction with fibular autograft alone did not produce graft fractures (Pieroh et al., 2016). The grafts could transmit stress through self-deformation, while there was a disparity in the elastic modulus between the metal and bone, which made stress concentrated. Therefore, remodeling the fibrous connection of the pubic symphysis such that it maintains a certain micro-movement is the key to achieve biological reconstruction. A flexible reconnection of the pubic symphysis combined with a rigid structure to provide muscle attachment points seems to be an ideal solution. Based on previous work demonstrating that 3D-printed porous structures could support soft tissue ingrowth (Guder et al., 2021; Xu et al., 2022), we infer that by relying on ordered micromotion during ambulation and tension–stress effect (Pilliar et al., 1986), the newborn strong, well-organized fibrous tissue could mimic native arcuate ligaments after elastic reconstruction (Murphy et al., 2021), albeit lacking histological verification.

In cases where the remaining ipsilateral pubis–ischial ramus is sufficient to attach prostheses, it remains unanimous to reconstruct region III in different centers. The integrated mega-prosthesis containing the pubis–ischia ramus has a complex spatial structure, and the arc-shaped arcuate line in particular requires excessive soft tissue exposure during placement. Obstruction of the iliopsoas muscle and neurovascular bundle aggravates the problem. In the following cases, we have tried to use the modular region III prosthetic connector to simplify the installation process. Moreover, the dismountable module of this site could minimize trauma of potential revision surgery.

We acknowledge the following shortcomings in this study. First, the FE model was simplified for the reduction of calculation. The analysis of the real biomechanical properties would require the inclusion of bones, cartilages, ligaments, and muscles. Stress in the region of contact changes in proportion to the cartilage thickness (Yang et al., 2020). It is also challenging to assess the extent of surrounding soft tissue after resection. Second, peak stress has been much lower than tensile strength and yield strength of the titanium alloy in this study. Gait analysis with a motion capture system should be incorporated into our future research. The biomechanical properties of the sitting position and the dynamic forces produced by the process of standing from a seated position would also be part of the work. Third, the porous interface has not been modeled. However, with the graded increase of the elastic modulus, force is expected to be transferred more smoothly.

## 5 Conclusion

The integrated lumbosacral mass *via* the bilateral rod–screw system and the novel sacral tray structure cooperated efficiently to alleviate the shear force. The instability in the absence of region III reconstruction could be compensated by the reinforcement of the posterior ring. The stability of the reconstructive system was expected to be longer with osseointegration achieved at the bone–prosthesis interface. The preliminary application in a small cohort manifested satisfactory function recovery and controllable complications. Further objective investigation on the durability and rate of long-term complications is required before introducing the system into routine clinical practice.

## Data Availability

The original contributions presented in the study are included in the article/supplementary material; further inquiries can be directed to the corresponding authors.

## References

[B1] Anderson A. E. Peters C. L. Tuttle B. D. Weiss J. A. (2005). Subject-specific finite element model of the pelvis: Development, validation and sensitivity studies. J. Biomechanical Eng. 127 (3), 364–373. 10.1115/1.1894148 16060343

[B2] Bergmann G. Deuretzbacher G. Heller M. Graichen F. Rohlmann A. Strauss J. (2001). Hip contact forces and gait patterns from routine activities. J. Biomechanics 34 (7), 859–871. 10.1016/s0021-9290(01)00040-9 11410170

[B3] Dong Y. Hu H. Zhang C.-Q. (2014). Biomechanical study of modular hemipelvic endoprosthesis for Type I-IV defect of pelvic tumor. Chin. J. Cancer Res. = Chung-kuo Yen Cheng Yen Chiu 26 (4), 431–436. 10.3978/j.issn.1000-9604.2014.08.13 25232216 PMC4153922

[B4] Eastman J. G. Krieg J. C. Routt M. L. Jr. (2016). Early failure of symphysis pubis plating. Injury 47 (8), 1707–1712. 10.1016/j.injury.2016.05.019 27282685

[B5] Enneking W. F. Dunham W. K. (1978). Resection and reconstruction for primary neoplasms involving the innominate bone. J. bone Jt. Surg. Am. volume 60 (6), 731–746. 10.2106/00004623-197860060-00002 701308

[B6] Enneking W. F. Dunham W. Gebhardt M. C. Malawar M. Pritchard D. J. (1993). A system for the functional evaluation of reconstructive procedures after surgical treatment of tumors of the musculoskeletal system. Clin. Orthop. Relat. Res. 286 (286), 241–246. 10.1097/00003086-199301000-00035 8425352

[B7] Esposito C. I. Carroll K. M. Sculco P. K. Padgett D. E. Jerabek S. A. Mayman D. J. (2018). Total hip arthroplasty patients with fixed spinopelvic alignment are at higher risk of hip dislocation. J. Arthroplasty 33 (5), 1449–1454. 10.1016/j.arth.2017.12.005 29310920

[B8] Guder W. K. Hardes J. Nottrott M. Podleska L. E. Streitburger A. (2021). Highly cancellous titanium alloy (TiAl6V4) surfaces on three-dimensionally printed, custom-made intercalary tibia prostheses: Promising short- to intermediate-term results. J. Pers. Med. 11 (5), 351. 10.3390/jpm11050351 33924789 PMC8145557

[B9] Hammer N. Scholze M. Kibsgård T. Klima S. Schleifenbaum S. Seidel T. (2019). Physiological *in vitro* sacroiliac joint motion: A study on three-dimensional posterior pelvic ring kinematics. J. Anat. 234 (3), 346–358. 10.1111/joa.12924 30536830 PMC6365483

[B10] Iqbal T. Shi L. Wang L. Liu Y. Li D. Qin M. (2017). Development of finite element model for customized prostheses design for patient with pelvic bone tumor. Proc. Inst. Mech. Eng. H. 231 (6), 525–533. 10.1177/0954411917692009 28639517

[B11] Li X. Ji T. Huang S. Wang C. Zheng Y. Guo W. (2020). Biomechanics study of a 3D printed sacroiliac joint fixed modular hemipelvic endoprosthesis. Clin. Biomech. (Bristol, Avon) 74, 87–95. 10.1016/j.clinbiomech.2020.02.014 32146381

[B12] Liang H. Ji T. Zhang Y. Wang Y. Guo W. (2017). Reconstruction with 3D-printed pelvic endoprostheses after resection of a pelvic tumour. bone & Jt. J. 99-B (2), 267–275. 10.1302/0301-620X.99B2.BJJ-2016-0654.R1 28148672

[B13] Liu D. Hua Z. Yan X. Jin Z. (2016). Biomechanical analysis of a novel hemipelvic endoprosthesis during ascending and descending stairs. Proc. Inst. Mech. Eng. H. 230 (10), 962–975. 10.1177/0954411916663970 27587536

[B14] Liu D. Jiang J. Wang L. Liu J. Jin Z. Gao L. (2019). *In vitro* experimental and numerical study on biomechanics and stability of a novel adjustable hemipelvic prosthesis. J. Mech. Behav. Biomed. Mater. 90, 626–634. 10.1016/j.jmbbm.2018.10.036 30500700

[B15] Murphy B. Thillainadesan T. Robinson K. Clarke A. Choong P. (2021). Case report: Reconstruction after anterior pubic hemipelvectomy. Front. Surg. 8, 585600. 10.3389/fsurg.2021.585600 34095198 PMC8177695

[B16] Otsuki B. Okamoto T. Fujibayashi S. Sakamoto A. Toguchida J. Murata K. (2021). Rigid reconstruction with periacetabular multiple screws after the resection of malignant pelvic tumours involving the sacroiliac joint. Int. Orthop. 45 (7), 1793–1802. 10.1007/s00264-021-05096-0 34086124

[B17] Peng W. Zheng R. Wang H. Huang X. (2020). Reconstruction of bony defects after tumor resection with 3D-printed anatomically conforming pelvic prostheses through a novel treatment strategy. BioMed Res. Int. 2020, 1–16. 10.1155/2020/8513070 PMC7723494 33335928

[B18] Pieroh P. Spindler N. Langer S. Josten C. Böhme J. (2016). A double-barrelled fibula graft restoring pelvic stability after late posterior ring instability related to a surgical treated osteitis pubis: A case report. Archives Orthop. Trauma Surg. 136 (1), 47–53. 10.1007/s00402-015-2355-y 26506827

[B19] Pilliar R. M. Lee J. M. Maniatopoulos C. (1986). Observations on the effect of movement on bone ingrowth into porous-surfaced implants. Clin. Orthop. Relat. Res. 208 (208), 108–113. 10.1097/00003086-198607000-00023 3720113

[B20] Ricci P. L. Maas S. Gerich T. Kelm J. (2020). Influence of pubic symphysis stiffness on pelvic load distribution during single leg stance. Int. J. Numer. Method Biomed. Eng. 36 (4), e3319. 10.1002/cnm.3319 32017442

[B21] Shen Y. W. Yang Y. Liu H. Qiu Y. Li M. Ma L. T. (2022). Biomechanical evaluation of intervertebral fusion process after anterior cervical discectomy and fusion: A finite element study. Front. Bioeng. Biotechnol. 10, 842382. 10.3389/fbioe.2022.842382 35372323 PMC8969047

[B22] Singh V. A. Elbahri H. Shanmugam R. (2016). Biomechanical analysis of a novel acetabulum reconstruction technique with acetabulum reconstruction cage and threaded rods after type II pelvic resections. Sarcoma 2016, 1–7. 10.1155/2016/8627023 PMC4906208 27340368

[B23] Sun W. Li J. Li Q. Li G. Cai Z. (2011). Clinical effectiveness of hemipelvic reconstruction using computer-aided custom-made prostheses after resection of malignant pelvic tumors. J. Arthroplasty 26 (8), 1508–1513. 10.1016/j.arth.2011.02.018 21477973

[B24] Tseng K.-Y. Lin K.-C. Yang S.-W. (2022). The radiographic outcome after plating for pubic symphysis diastasis: Does it matter clinically? Archives Orthop. Trauma Surg. 10.1007/s00402-022-04411-7 PMC10030392 35278092

[B25] Wang B. Sun P. Xie X. Wu W. Tu J. Ouyang J. (2015). A novel combined hemipelvic endoprosthesis for peri-acetabular tumours involving sacroiliac joint: A finite element study. Int. Orthop. 39 (11), 2253–2259. 10.1007/s00264-015-2891-7 26183143

[B26] Wang H. Su K. Su L. Liang P. Ji P. Wang C. (2018). The effect of 3D-printed Ti6Al4V scaffolds with various macropore structures on osteointegration and osteogenesis: A biomechanical evaluation. J. Mech. Behav. Biomed. Mater. 88, 488–496. 10.1016/j.jmbbm.2018.08.049 30223212

[B27] Wang B. Sun P. Yao H. Tu J. Xie X. Ouyang J. (2019a). Modular hemipelvic endoprosthesis with a sacral hook: A finite element study. J. Orthop. Surg. Res. 14 (1), 309. 10.1186/s13018-019-1338-z 31511034 PMC6739965

[B28] Wang B. Zou C. Hu X. Tu J. Yao H. Yin J. (2019b). Reconstruction with a novel combined hemipelvic endoprosthesis after resection of periacetabular tumors involving the sacroiliac joint: A report of 25 consecutive cases. BMC cancer 19 (1), 861. 10.1186/s12885-019-6049-7 31470808 PMC6716888

[B29] Wang J. Min L. Lu M. Zhang Y. Wang Y. Luo Y. (2020). What are the complications of three-dimensionally printed, custom-made, integrative hemipelvic endoprostheses in patients with primary malignancies involving the acetabulum, and what is the function of these patients? Clin. Orthop. Relat. Res. 478 (11), 2487–2501. 10.1097/CORR.0000000000001297 32420722 PMC7594920

[B30] Wang H. Tang X. Ji T. Yan T. Yang R. Guo W. (2021). Risk factors for early dislocation of the hip after periacetabular tumour resection and endoprosthetic reconstruction of the hemipelvis. Bone & Jt. J. 103-B (2), 382–390. 10.1302/0301-620X.103B2.BJJ-2020-0928.R1 33517736

[B31] Wang M. Liu T. Xu C. Liu C. Li B. Lian Q. (2022). 3D-printed hemipelvic prosthesis combined with a dual mobility bearing in patients with primary malignant neoplasm involving the acetabulum: Clinical outcomes and finite element analysis. BMC Surg. 22 (1), 357. 10.1186/s12893-022-01804-8 36203147 PMC9541076

[B32] Wu J. Xie K. Luo D. Wang L. Wu W. Yan M. (2021). Three-dimensional printing-based personalized limb salvage and reconstruction treatment of pelvic tumors. J. Surg. Oncol. 124 (3), 420–430. 10.1002/jso.26516 34086993

[B33] Xu S. Guo Z. Shen Q. Peng Y. Li J. Li S. (2022). Reconstruction of tumor-induced pelvic defects with customized, three-dimensional printed prostheses. Front. Oncol. 12, 935059. 10.3389/fonc.2022.935059 35847863 PMC9282862

[B34] Yang J. Zhao G. Xu H. Wang F. (2020). Three-Dimensional finite element analysis of the effects of ligaments on human sacroiliac joint and pelvis in two different positions. J. Biomechanical Eng. 142 (8), 081007. 10.1115/1.4046361 32060536

[B35] Zanetti E. M. Bignardi C. Audenino A. L. (2012). Human pelvis loading rig for static and dynamic stress analysis. Acta Bioeng. Biomechanics 14 (2), 61–66. 22793862

[B36] Zhang Y. Tang X. Ji T. Yan T. Yang R. Yang Y. (2018). Is a modular pedicle-hemipelvic endoprosthesis durable at short term in patients undergoing enneking type I + II tumor resections with or without sacroiliac involvement? Clin. Orthop. Relat. Res. 476 (9), 1751–1761. 10.1007/s11999.0000000000000121 30794212 PMC6259779

[B37] Zhang Y. Min L. Lu M. Wang J. Wang Y. Luo Y. (2021). Three-dimensional-printed customized prosthesis for pubic defect: Clinical outcomes in 5 cases at a mean follow-up of 24 months. BMC Musculoskelet. Disord. 22 (1), 405. 10.1186/s12891-021-04294-6 33941162 PMC8091684

[B38] Zhao X. Xiao J. Sun Y. Zhu Z. Xu M. Wang X. (2018). Novel 3D printed modular hemipelvic prosthesis for successful hemipelvic arthroplasty: A case study. J. Bionic Eng. 15 (6), 1067–1074. 10.1007/s42235-018-0094-9

[B39] Zhou R. Xue H. Wang J. Wang X. Wang Y. Zhang A. (2022). Improving the stability of a hemipelvic prosthesis based on bone mineral density screw channel and prosthesis optimization design. Front. Bioeng. Biotechnol. 10, 892385. 10.3389/fbioe.2022.892385 35706507 PMC9189365

[B40] Zhu D. Fu J. Wang L. Guo Z. Wang Z. Fan H. (2021). Reconstruction with customized, 3D-printed prosthesis after resection of periacetabular Ewing's sarcoma in children using "triradiate cartilage-based" surgical strategy:a technical note. J. Orthop. Transl. 28, 108–117. 10.1016/j.jot.2020.12.006 PMC8022806 33868923

